# Associations between Stress and Physical Activity in Korean Adolescents with Atopic Dermatitis Based on the 2018–2019 Korea Youth Risk Behavior Web-Based Survey

**DOI:** 10.3390/ijerph17218175

**Published:** 2020-11-05

**Authors:** Sunga Kong, Jaisun Koo, Seung Kil Lim

**Affiliations:** 1 Department of Clinical Research Design and Evaluation, SAIHST, Sungkyunkwan University, Seoul 06351, Korea; sunga00kong@gmail.com; 2 Patient-Centered Outcomes Research Institute, Samsung Medical Center, Seoul 06351, Korea; 3 Da Vinci College of General Education, Chung-Ang University, Seoul 06974, Korea; 4 Department of Exercise Prescription, Dongshin University, Naju, Jeonnam 58245, Korea

**Keywords:** atopic dermatitis, stress, physical activity, exercise, youth

## Abstract

This study aimed to confirm the associations between stress and physical activity (PA) in Korean adolescents with atopic dermatitis (AD) based on data from the 2018–2019 Korea Youth Risk Behavior Web-Based Survey. The AD groups were divided into adolescents who were not diagnosed with AD, adolescents who were diagnosed with AD more than one year ago, and adolescents who were diagnosed with AD within one year. We defined the regular PA group and the non-PA group using the American College of Sports Medicine guidelines for children and adolescents: moderate to vigorous PA ≥5 times per week, including vigorous PA ≥3 days per week and muscle strengthening exercises ≥3 times per week. We performed logistic regression analysis to calculate the stress odds ratio (OR) and 95% confidence interval (CI) by group using model 1, adjusted for age, sex, and body mass index, and model 2, additionally adjusted for drinking, smoking, economic statuses, academic achievement, asthma, and rhinitis. In the group diagnosed with AD within one year, stress was 41% and 32% higher according to models 1 (1.41 (1.31–1.52)) and 2 (1.34 (1.20–1.50)), respectively. However, the stress OR was 30% lower in adolescents who completed regular PA than in the non-PA group (model 1: 0.71 (0.58–0.87); model 2: 0.68 (0.57–0.84)), even if diagnosed with AD within one year. In conclusion, the stress of adolescents with AD was significantly higher than that of adolescents without AD. The stress was significantly lower in the group with regular PA, and it was more robust in adolescents diagnosed with AD within one year.

## 1. Introduction

Atopic dermatitis (AD) is a disease characterized by repeated cycles of exacerbation and improvement of itchy eczema [1]. Among adolescents, AD is one of the most common chronic diseases, along with asthma and rhinitis [2]. The incidence of AD in children has increased two- to three-fold over the past 30 years [3,4], with an incidence of more than 20% among children and adolescents worldwide [5]. In South Korea, the incidence of AD among children and adolescents is currently 13.5% and increasing [6]. The main symptoms of AD include itching and hypersensitivity to external stimuli and allergens, and these symptoms are known to worsen in adolescence [7]. Although the etiology of AD is still unclear, studies have reported that it is caused by an interaction between genetic, environmental, and immunological factors and a dysfunction in the skin barrier [8].

AD degrades quality of life by affecting an individual’s sleep, emotional and mental health, physical activity (PA), and social functioning [9]. In particular, adolescents are known to demonstrate problems in establishing a healthy body image; they have low perceived happiness and high stress, as they undergo esthetic and functional skin changes caused by chronic and repeated inflammatory reactions [10]. Furthermore, adolescents experience negative social and psychological changes, such as depression and anxiety [11,12]. A prior study reported higher suicidal ideation (44%) and suicide attempts (36%) among patients with AD compared to their non-AD counterparts [13]. A study of data from the Korea Youth Risk Behavior Web-Based Survey (KYRBWS) VI reported that 46% of adolescents with a high level of stress and 21% of adolescents with a moderate level of stress suffer from AD [14]. In light of these studies, AD may not only induce stress, but it may also be caused by stress [11]. Therefore, the stress level in youths with AD is an important factor in terms of the improvement of disease symptoms and treatment, as well as patient mental health.

Among various measures to manage mental health, regular PA is known to contribute to improving physical function and mental stress [15,16]. An animal study reported that moderate-intensity aerobic exercise improves the symptoms of AD through immunoregulation [17]. However, no human studies have been reported to date. Moreover, PA in patients with AD has both positive and negative aspects, as it can help improve AD by reducing stress [15,16], but can also exacerbate symptoms of AD by increasing body temperature and inducing perspiration [18]. Although studies in Korea and abroad have investigated the percentage of adolescents with AD who participate in PA [19,20], none have examined the relationship between stress and PA in adolescents with AD; hence, the impact of PA on stress in adolescents with AD remains unclear. Thus, it is necessary to investigate the relationship between stress and PA in adolescents with AD. This study aimed to investigate the risk of stress in adolescents with AD and the association between stress and regular PA by using data from the KYRBWS.

## 2. Methods

### 2.1. Study Participants

Raw data from the 2018 (14th) and 2019 (15th) KYRBWS, a government-approved statistical survey examining the health behaviors of middle and high school students in Korea, were analyzed in this study. The raw data were gathered from the Korea Centers for Disease Control and Prevention website after submitting an application outlining the purpose and plan of use and obtaining approval. The KYRBWS was conducted on 7th to 12th graders in 800 middle and high schools in Korea, selected by stratifying the population, allocating the sample, and extracting the sample. A total of 60,040 out of 62,823 (95.6%) participants completed the KYRBWS-14, and 57,303 out of 60,100 (95.3%) participants completed the KYRBWS-15. Data from these participants (*N* = 117,343) were used in the final analysis.

### 2.2. Measurements

Participants who answered “No” to the question, “Have you ever been diagnosed with atopic dermatitis (eczema or congenital fever) by a physician?” were included in the non-AD group. Those who answered “Yes” to the same question were additionally asked, “Have you been diagnosed with atopic dermatitis (eczema or congenital fever) by a physician in the past 12 months?” to further divide AD participants into the AD ≥1 year and AD <1 year groups.

For stress, those who chose “extremely high,” “high,” or “moderate” to the question, “How is your day-to-day stress level?” were considered to have stress, whereas those who chose “low” or “none” were considered to have no stress.

To define regular PA, participants were asked, “How many days in the past 7 days have you engaged in PA (all types) that increases your heart rate above the normal rate or makes you short of breath for at least 60 min a day?” and were divided into the “≥5 days” or “<5 days” categories. Participants were also asked, “How many days in the past 7 days have you engaged in vigorous PA that makes you extremely short of breath or sweat for at least 20 min a day?” and were divided into the “≥3 days” or “<3 days” categories. Participants were also asked, “How many days in the past 7 days have you engaged in muscle strengthening exercises, such as push-ups, sit-ups, lifting weights, using dumbbells, pull up bar, and parallel bar?” and were divided into the “≥3 days” or “<3 days” categories [21].

For physical characteristics, participants’ age, sex, and body mass index (BMI; kg/m^2^) were examined. Smoking status was determined using the yes-or-no question, “Have you ever smoked at least one cigarette?” and drinking status was determined using the yes-or-no question, “Have you ever had at least one shot of alcohol?” Economic status was determined using the question, “What is your household’s economic status?” The responses were grouped into “high” and “middle-high,” or into “middle,” “middle-low,” and “low.” Academic achievement was determined using the question, “How were your school grades in the past 12 months?” The responses were grouped into “high” and “middle-high,” or into “middle,” “middle-low,” and “low.”

### 2.3. Statistical Analysis

All statistical analyses were performed using STATA version 15.0 (STATA Corp., College Station, TX, USA). A complex sample design was used to compute representative values for the Korean adolescent population. The physical characteristics according to the AD group (non-AD, AD ≥1 year, and AD <1 year) were compared using the mean, standard error, and percentage within each group (%). The participants’ characteristics according to AD groups were examined by the *F*-test of the average between AD groups (all AD diagnoses) compared to the non-AD group using a complex samples regression. Differences in smoking status, drinking status, economic status, academic achievement, moderate and vigorous physical activity (MVPA), and muscle strengthening exercises among the AD groups were analyzed with chi-square tests. The stress odds ratio (OR) according to the AD group was compared using multiple logistic regression. The ORs and 95% confidence intervals (CI) for the AD groups were presented in models 1 and 2 based on adjustment. Model 1 was adjusted for age, sex, and obesity (BMI <18.5, 18.5–22.9, or ≥23), and model 2 was additionally adjusted for drinking status, smoking status, economic status, academic achievement, asthma, and rhinitis. Furthermore, the stress OR according to AD and PA was analyzed using the same method. Statistical significance was set at α = 0.05 for all analyses.

## 3. Results

### 3.1. Participant’s Characteristics According to AD Diagnosis

The participant characteristics of the non-AD, AD ≥1 year, and AD <1 year groups are described in Table 1. A total of 8099 out of 117,343 (6.8%) participants had been diagnosed with AD within one year. The AD group was older and had a higher BMI than the non-AD group (*p* < 0.001). The AD <1 year group had a higher percentage of respondents who reported smoking and drinking but lower MVPA and muscle strengthening exercises compared to the non-AD group (Table 1).

### 3.2. Stress OR According to AD and PA

Compared to the non-AD group, the AD <1 year group had a significantly higher OR for stress, at 1.41 (1.31–1.52) in model 1 and 1.34 (1.20–1.50) in model 2 (*p* < 0.001). However, the AD ≥1 year group only had a significantly higher OR in model 1, at 1.17 (1.11–1.23), but not in model 2, at 1.06 (0.98–1.14) (Table 2).

### 3.3. Stress OR According to AD and PA

The stress OR among the AD groups was compared according to PA. Those who engaged in regular PA had a significantly lower stress OR than in the non-AD group, with 0.79 (0.75–0.84) in model 1 and 0.76 (0.72–0.81) in model 2 (*p* < 0.001). The OR for stress was also significantly lower in the AD ≥1 year group, with 0.83 (0.72–0.97) in model 1 and 0.80 (0.70–0.93) in model 2 (*p* < 0.05). Finally, the OR for stress was also significantly lower in the AD <1 year group, with 0.71 (0.58–0.87) in model 1 and 0.68 (0.57–0.84) in model 2 (*p* < 0.001) (Table 3).

## 4. Discussion

This study analyzed data from the KYRBWS obtained from the Korean adolescent population. The risks of stress in adolescents diagnosed with AD within one year were 41% and 34% higher than in non-AD adolescents in the two adjusted models, respectively. However, among those diagnosed with AD, adolescents who engaged in regular PA had a significantly lower stress risk compared to the non-PA adolescent group, and these results were more robust among adolescents diagnosed with AD within one year.

In this study, we showed that stress was significantly higher in the AD group than in the non-AD group. These results are consistent with those of prior cross-sectional [14,22] and longitudinal studies [23], where AD and stress were reported to be associated. Park and Kim (2016) observed that the risk for severe stress was twice as high in adolescents with AD compared to their healthy counterparts [22], and Kwon (2013) reported that male adolescents with severe stress had a 46% higher risk of AD [14]. Our results also demonstrated that AD and stress are associated, supporting previous findings. In general, AD symptoms and mental stress affect one another in a vicious cycle. Some studies have reported that stress affects the development and progression of AD and exacerbates its symptoms by stimulating the hypothalamic–pituitary–adrenal axis [24]. According to recent research, the evaluation of psychoneuroimmunologic factors and emotional stress is an important component of treatment, in addition to interventions, to improve mental health and reduce stress for patients with AD, as well as to improve patients’ health and to markedly improve skin symptoms [24]. Thus, health behavior-related factors that may contribute to improving stress in adolescents with AD should be considered.

In this study, regular PA was associated with a lower risk of stress compared to the non-PA group. The results from previous studies confirmed that regular PA can help improve physical and psychological health among children and adolescents [25,26]. In a recently reported model- and data-based research results, it was reported that PA is related to adolescent mental health among young British adolescents [27]. In that study, regular PA was linked with better mental health, characterized by higher self-esteem and fewer internalizing problems (*β* = −0.24; 95% CI, −0.27 to −0.20). As shown in the systematic review by Mücke et al., high PA reduces stress-related cortisol and heart rate responsiveness, even when participants were exposed to high stress levels [28]. They reviewed smaller increases in anxiety and smaller decreases in calmness in participants with regular PA. In our study, even the group diagnosed with AD within one year also demonstrated a 30% lower risk of stress when they engaged in regular PA, compared to the non-PA group. These results are consistent with those of previous studies demonstrating that exercise and PA help relieve mental stress in adults, adolescents, and patients with allergic diseases, such as asthma [29,30,31]. A prior study on 147 adolescents reported that a 10-week vigorous-intensity exercise program (30 min/session, two sessions/week) significantly reduced stress [30]. Furthermore, a study that administered a PA intervention in a school environment for low-income adolescents reported that the intervention improved the students’ cardiopulmonary function (VO_2max_) by 8.5% and reduced anxiety symptoms by 13.7% [31]. In addition, an MVPA intervention program, including psychological management, was also reported to reduce stress in undergraduate students [32]. In general, stress is associated with mental health problems, such as depression and suicide, and it may affect sleep, cardiovascular diseases, and immune system dysfunction [33]. In fact, young adults with an increased risk of mental disorder symptoms or a high incidence of mental disorders may develop serious social and occupational dysfunction [34]. Therefore, it is important to promote PA in adolescents with AD, who are already at high risk of stress, to manage their mental health.

In this study, we could not examine the exact effects of regular PA on adolescents’ allergic immune responses. However, we observed that regular PA was positively associated with psychological stress in the Korean adolescent population. Some previous studies have reported that PA is associated with AD symptoms, and that patients with severe AD participate in approximately 50% less PA (adjusted OR, 0.45; 95% CI, 0.28–0.73) [35]. In general, MVPA causes shortness of breath or sweat. In a recent study, through metabolomics analysis of sweat in patients with AD, it was shown that the glucose concentration in sweat increases with the severity of the disease [36]. High glucose levels in sweat can delay the repair of the damaged skin barrier and promote itching. Furthermore, since patients with AD can have a warm sensation on their skin and sweat caused by PA can actually induce itching, PA may exacerbate the symptoms of AD [18]. The results of this study do not reveal whether AD patients’ symptoms worsen due to sweating after PA because it was a cross-sectional study of the Korea Youth Risk Behavior Web-Based Survey. Therefore, the causal relationship between AD symptoms and sweat caused by PA in adolescents with AD is unknown. Thus, future studies should examine both PA promotion and symptom improvement in patients with AD.

This study has a few limitations. First, this study used nationally representative data obtained via an online survey, and thus, we could not obtain information about AD treatment history and severity. However, one strength of this study is that the study data were extracted from a sample of more than 100,000 individuals by a government agency. Second, this was a cross-sectional study; therefore, the causality between stress and PA in adolescents with AD could not be determined. However, we specified the time of diagnosis to within one year, and the survey asked about PA within the past week, which enabled an understanding of the association between the current level of stress and PA. Third, the KYRBWS used multiple-choice questions, so the exact amount of PA could not be determined. However, the survey contained separate questions for moderate PA, vigorous PA, and muscle strengthening exercises, so we were able to distinguish intensity and form of exercise.

## 5. Conclusions

In this study, adolescents with AD had a higher stress OR than adolescents without; in particular, those who had been diagnosed with AD within one year had a 30% higher risk. However, adolescents with AD who regularly engaged in MVPA and muscle strengthening exercises had significantly lower stress. Thus, it would be beneficial to emphasize the importance of PA in managing mental health for adolescents with AD who have a high risk of stress.

## Figures and Tables

**Table 1:** Participant characteristics by atopic dermatitis diagnosis.

	Non-AD (*n* = 89,863)	AD ≥ 1 year (*n* = 19,381)	AD < 1 year (*n* = 8099)	*p*-Value
Age, years	15.1 ± 0.0	15.3 ± 0.0 ^a^	15.2 ± 0.3 ^a,b^	<0.001
Male sex (%)	54.3	44.8	43.8	<0.001
Body mass index, kg/m^2^	21.3 ± 0.0	21.4 ± 0.0	21.6 ± 0.1 ^a,b^	<0.001
Grade (%)				<0.001
Middle school	48.1	43.5	45.3	
High school	52.9	56.5	54.7	
Smoking status, yes (%)	13.9	12.5	16.5	<0.001
Alcohol status, yes (%)	40.3	42.3	43.3	<0.001
Household income (%)				<0.001
High or medium-high	40.7	38.9	39.0	
Medium, medium-low, or low	59.3	61.1	61.0	
Academic achievement (%)				<0.001
High or medium-high	38.0	40.6	37.7	
Medium, medium-low, or low	62.0	59.4	62.3	
Moderate PA (%)				<0.001
<5 days per week	85.2	87.7	86.0	
≥5 days per week	14.8	12.3	14.0	
Vigorous PA (%)				<0.001
<3 days per week	64.2	68.4	66.3	
≥3 days per week	35.8	31.5	33.7	
Muscle strengthening exercises (%)				<0.001
No or 1 time per week	76.5	80.8	78.5	
≥3 days per week	23.5	19.2	21.5	
Regular PA (%)	8.7	6.9	7.9	<0.001

Data are presented as mean ± standard error, percent (%). Non-AD: The group of adolescents not diagnosed with atopic dermatitis (AD). AD ≥1 year: The group of adolescents diagnosed with AD more than one year ago. AD <1 year: The group of adolescents diagnosed with AD within one year. PA: Physical activity. Regular physical activity (%): Moderate and vigorous physical activity (MVPA) ≥5 times per week, including vigorous PA ≥3 days per week and muscle strengthening exercises ≥3 times per week. ^a^ *p* < 0.05 compared to the non-atopic dermatitis group. ^b^ *p* < 0.05 compared to the AD group.

**Table 2:** Odds ratios of stress status by atopic dermatitis diagnosis.

	Non-AD (*n* = 89,863)	AD ≥ 1 year (*n* = 19,381)	AD < 1 year (*n* = 8099)	*p*-Value
^a^ Model 1 OR (95% CI)	Reference	1.17 (1.11–1.23) *	1.41 (1.31–1.52) *	<0.001
^b^ Model 2 OR (95% CI)	Reference	1.06 (0.98–1.14)	1.34 (1.20–1.50) *	<0.001

OR: Odds ratio; CI: Confidence interval. ^a^ Model 1: Adjusted for age, sex, and body mass index (<18.5, 18.5–22.9, or ≥23 kg/m^2^). ^b^ Model 2: Further adjusted for household income (<medium-high or ≥medium-high), academic achievement (<medium-high or ≥medium-high), smoking status, alcohol status, and other diseases (asthma or allergic rhinitis). Non-AD: The group of adolescents not diagnosed with atopic dermatitis (AD). AD ≥1 year: The group of adolescents diagnosed with AD more than one year ago. AD <1 year: The group of adolescents diagnosed with AD within one year. * Significantly different from the reference group (*p* < 0.05).

**Table 3:** Odds ratios of stress status by atopic dermatitis diagnosis and physical activity.

	Non-AD (*n* = 89,863)	AD ≥ 1 year (*n* = 19,381)	AD < 1 year (*n* = 8099)
^a^ Model 1 OR (95% CI)			
Non-PA	Reference	Reference	Reference
Regular PA	0.79 (0.75–0.84) *	0.83 (0.72–0.97) *	0.71 (0.58–0.87) *
*p*-Value	<0.001	0.015	0.001
^b^ Model 2 OR (95% CI)			
Non-PA	Reference	Reference	Reference
Regular PA	0.76 (0.72–0.81) *	0.80 (0.70–0.93) *	0.68 (0.57–0.84) *
*p*-Value	<0.001	0.003	<0.001

OR: Odds ratio; CI: Confidence interval; PA: Physical activity. ^a^ Model 1: Adjusted for age, sex, and body mass index (<18.5, 18.5–22.9, or ≥23 kg/m^2^). ^b^ Model 2: Further adjusted for household income (<medium-high or ≥medium-high), academic achievement (<medium-high or ≥medium-high), smoking status, alcohol status, and other diseases (atopic dermatitis or allergic rhinitis). Non-AD: The group of adolescents not diagnosed with atopic dermatitis (AD). AD ≥1 year: The group of adolescents diagnosed with AD more than one year ago. AD <1 year: The group of adolescents diagnosed with AD within one year. Regular physical activity (%): MVPA ≥5 times per week, including vigorous PA ≥3 days per week and muscle strengthening exercises ≥3 times per week. * Significantly different from the reference group (*p* < 0.05).
